# A Scoping Review of Interventions for the Treatment of Eco-Anxiety

**DOI:** 10.3390/ijerph18189636

**Published:** 2021-09-13

**Authors:** Pauline Baudon, Liza Jachens

**Affiliations:** Psychology, Sociology and Professional Counselling, Webster University, 1293 Geneva, Switzerland; pauline.baudon@gmail.com

**Keywords:** eco-anxiety, climate anxiety, climate change, therapeutic approaches, psychological interventions, scoping review

## Abstract

As climate change worsens and public awareness of its grave impact increases, individuals are increasingly experiencing distressing mental health symptoms which are often grouped under the umbrella term of eco-anxiety. Clear guidance is needed to enable mental health professionals to make informed choices of appropriate interventions and approaches in their eco-anxiety treatment plans. A scoping review was conducted to examine the current understanding of eco-anxiety and related intervention options and recommendations. The review included 34 records, 13 of which reflected specific psychological approaches. A thematic analysis of the content of the selected records yielded five major themes across interventions for individual and group treatment of eco-anxiety: practitioners’ inner work and education, fostering clients’ inner resilience, encouraging clients to take action, helping clients find social connection and emotional support by joining groups, and connecting clients with nature. Recommendations for treatment plans are to focus on holistic, multi-pronged, and grief-informed approaches that include eco-anxiety focused group work.

## 1. Introduction

The impact of climate change, the “unfamiliar, human-induced changes in atmosphere and depletion in biodiversity and other natural systems” [1] (p. 27) manifesting through significant changes in the planet’s temperature, wind patterns, rainfall, and other measures of climate [2], is becoming more and more apparent. This impact is both actual and predicted [3,4]. Indeed, current scientific consensus states that global warming is occurring and will continue to occur, with profound effects on public and environmental health [5]. Individuals increasingly wrestle with visions of a future life with more extreme weather events, higher temperatures, increased respiratory illness, changes in water and food availability, mass displacement of populations, and the pain of imagining the devastation and loss of familiar environments [5,6,7]. In fact, the 2019 United Nations Climate Summit in New York named climate change as the defining issue of our time [8].

Public awareness of the effects of climate change is rising due to growing media coverage and the release of alarming reports and warnings by major organizations like the United Nations and the World Health Organization [6,9]. The Yale Program on Climate Change Communication found in 2018 that “69% of Americans worry about global warming, and 49% believe it will harm them personally” [10] (p. 13). Across the world, survey data from the Australian youth mental health organization ReachOut revealed that four out of five students felt somewhat or very anxious about climate change. Half of the students further reported that they experienced these emotions on a weekly basis [11].

Research confirms that our collective sense of a looming climate change-related threat is taking a significant toll on our mental health [5]. Recent studies [12,13] point to a surge in the psychological distress associated with “awareness of the overarching problem humans face as a result of global climate change” [12] (p. 7) [13]. The International Psychoanalytical Association now names climate change as the greatest global health threat of the 21st century [14]. Though research on this specific form of distress is still in its infancy, the umbrella term of eco-anxiety, which is not yet listed in the DSM-5 (Diagnostic and Statistical Manual of Mental Disorders), is emerging as a key term.

The term is used to describe the emotional and mental states associated with heightened awareness of climate change and concurrent distress in the face of its threatening implications for the future [7]. The American Psychological Association describes it as a “chronic fear of environmental doom” [15] (p. 68). Psychologist Renee Lertzman refers to the experience as “environmental melancholia- a combined sense of primal loss and paralyzing powerlessness” (as quoted by Dockett [16], (p. 11)) while environmental philosopher and professor of sustainability Glenn Albrecht [17] categorizes it as a form of “psychoterratic illness…(an) earth-related mental illness where people’s mental wellbeing… is threatened by the severing of ‘healthy’ links between themselves and their home” (p. 95).

Though the term eco-anxiety certainly implies the presence of anxiety as a key symptom, individuals who suffer from it experience a constellation of emotions [18] including fear, anger, exhaustion, powerlessness, feelings of loss, helplessness [19,20], and even phobia and despair [21]. Lise Van Susteren, a psychiatrist specialized in the psychological effects of climate change, sees eco-anxiety as a form of “pre-traumatic stress disorder” (as cited in Kerecman Myers [22], (p. 3)), in which traumatic consequences are anticipated and felt before the event even takes place. Kaplan [23] has similarly explored the traumatizing power of anticipation in relation to climate change and the possibility of an emerging “Pre-Traumatic Stress Syndrome” (p. 81), including symptoms like flash-forwards, fear-induced dissociation, and nightmares.

The need for informed professionals certainly seems more pressing than ever, as the prevalence of eco-anxiety and demand for its treatment are on the incline. The UK-based Climate Psychology Alliance reported in 2019 that it was “inundated with requests for therapeutic support” [24] while US-based grief support organization The Good Grief Network, which coordinates support groups for eco-anxiety, reported that the last 6 months of 2019 saw a surge of interest in their activities “with branches popping up in half a dozen states” [24].

Many mental health professionals have called for an increase in awareness of and training for eco-anxiety [5,15,25,26]. Despite the apparent and increasing demand for therapeutic support and the projection that climate change will only continue to loom larger and larger in collective awareness, very little existing literature seems to address relevant and specific therapeutic interventions for eco-anxiety. It is therefore key to start assembling a clear picture of appropriate interventions and approaches to enable these professionals to make informed choices in their eco-anxiety treatment plans.

As scoping studies are particularly pertinent to topics with emerging evidence, a scoping review of the literature was undertaken to identify the breadth of the peer-reviewed literature on the topic of eco-anxiety. It is important to note that this review will focus on treatment for individuals experiencing the indirect distress and eco-anxiety related to the general threat posed by climate change, rather than distress experienced as the direct result of a natural disaster or any climate-change related weather event, which may include more of a focus on disaster resiliency and post-traumatic stress disorder [12].

Therefore, the main research questions is:

1. What is known from the existing published literature about interventions for the individual and group treatment of eco-anxiety?

The secondary research questions are:

2. What published literature is available from different psychological approaches/perspectives on interventions for the treatment of eco-anxiety?
3. How do different psychological approaches differ or concur on the interventions they propose for the treatment of eco-anxiety?
4. What published literature exists proving the efficacy of any of these interventions?

## 2. Methods

The scoping review method aims to “map key concepts, sources, and types of evidence that underpin a research area (…) when literature on a topic is being assembled for the first time, and (or) when the topic under investigation is complex or nonhomogeneous” [27] (p. 386). Our process within this scoping review followed Arksey and O’Malley’s five stage methodological framework [28]. Additionally, the review followed the PRISMA Extension for Scoping Reviews (PRISMA-ScR) checklist (http://www.prisma-statement.org/Extensions/ScopingReviews) (accessed on 12 July 2021).

After identifying the research questions, relevant records were selected. The literature search included English language articles (authors’ common language) from peer-reviewed journals and magazines, blog entries, and organization reports. Books and book chapters were excluded due to the time constraints of the study. No limitations were placed on the populations studied or intended. Records relevant to the aim of this study were identified through an explorative search on the following databases: Academic Search Complete, Business Source Complete, Military & Government Collection, APA PsycInfo, CINAHL with Full Text, SocINDEX with Full Text, Environment Complete, Humanities International Complete, Consumer Health Complete–EBSCOhost, Google Scholar, and ResearchGate, between 1 February 2019 and 23 June 2020. The specific search terms for the database search are listed in Appendix A.

**Figure 1:**
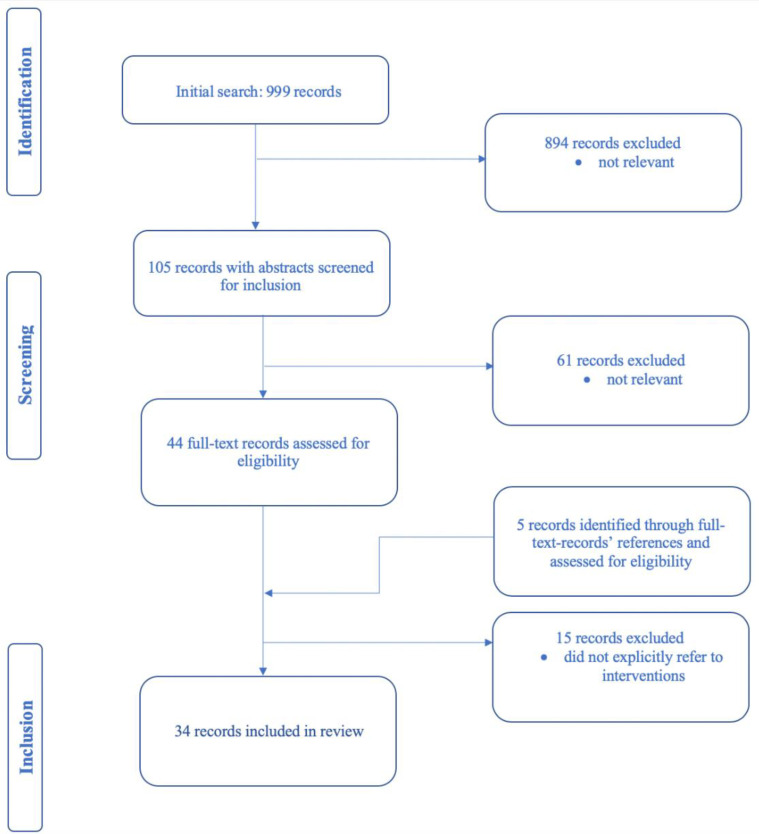
Records identification, screening and selection.

The process of selecting records for inclusion in the study relied on four steps (Figure 1). Firstly, an initial pool of records was identified through databases; secondly, the selected pool underwent assessment by title and abstract; thirdly, the full text of records chosen was assessed for eligibility and extra records were identified through the reference list of full-text records. Eligible records chosen through this fourth and final step were included in the review. The record search process was carried out by the author (P.B.) and reviewed by second author (L.J.).

Each article’s author(s), year of publication, journal or publisher, psychological approach, and proposed interventions were collected and entered in an Excel spreadsheet.

Records are organized in the alphabetical order of the psychological approaches they reflect. Central themes were identified during data analysis using Braun and Clarke’s [29] guidelines involving a process of inductive thematic content analysis. The authors ‘actively’ read the articles to find meaning and patterns in the data. Next, ‘codes’ were constructed, which are aspects of the data that appear relevant and interesting and refer to the most basic element of information that can be assessed in a meaningful way. The initial codes were then grouped into categories according to their similarities. Categories were organized into themes, described as data that show a patterned response or meaning within the data set. Two coders were used (authors) to achieve inter-rater reliability and to refine themes.

## 3. Results

The review included 34 records, 13 of which reflected specific psychological approaches. A thematic analysis of the content of the selected records yielded five major themes across interventions for individual and group treatment of eco-anxiety (Table 1).

Nine hundred and ninety-nine records were identified from the various database searches. After removing non-relevant literature, 34 records published between 2005 and 2019 were included in the review. Twenty of the records reviewed were conceptual or reflective papers [7,12,16,30,31,32,33,34,35,36,37,38,39,40,41,42,43,44,45,46], five were organization reports [13,15,47,48,49], three were mixed methods studies [26,50,51], three were magazine articles [10,52,53], one was a qualitative study [54], one was a chapter from a conference report [55], and one was a blog post [56].

There was a great variety in psychotherapeutic approaches. Seven records reflected or included a psychoanalytical approach, four records reflected or included an ecotherapy and ecopsychology approach, and two records reflected or included a Jungian depth psychology approach. There was one record of each of the following approaches: environmental education, social work/social psychology, conservation psychology, marriage and family therapy, counselling and narrative therapy, Gestalt approach, and an environmental science approach. Fourteen records did not claim to reflect any specific approach.

Of the 34 records included in the review, only four were empirical research studies [26,50,51,54], of which two directly evaluated interventions for eco-anxious individuals [50,54], one included practitioners’ accounts of approaches that have been successful in their treatment of eco-anxious individuals [26], and one did not evaluate any interventions for eco-anxiety but did propose some interventions [51]. Gillespie’s [54] mixed-methods group research found that a group process in which discussions of dreams related to eco-anxiety helped participants to bring up distressing experiences, process difficult emotions and feel more able to engage with issues related to climate change. Büchs et al.’s [50] mixed-methods assessment of individuals’ experience of Carbon Conversations group found that 50% of participants agreed or strongly agreed that taking part in Carbon Conversations helped them face their worries about climate change, and that many were especially appreciative of the space provided for expressing difficult and complex emotions on the topic. Like participants in Gillespie’s study, Carbon Conversation participants reported that the experience helped them to move into or stay engaged with ecologically motivated action and lifestyle changes. Seaman’s [26] mixed-methods study determined how often distress related to climate change emerged in the therapeutic setting and how practitioners handled it. Practitioners reported that they initially focused on displaying empathy and validations and asking questions about coping and self-care. In terms of which treatment modalities they found to be helpful to eco-anxious clients, they provided varied answers, including PTSD (Post Traumatic Stress Disorder) treatment, psychodynamic therapy, Motivational Interviewing, Cognitive Behavioral Therapy, and Mindfulness-Based Cognitive Behavioral Therapy [26]. No specific interventions involving these modalities were mentioned.

One of this review’s secondary research questions asked how different psychological approaches differed or concurred on the interventions they recommended for the treatment of eco-anxiety. The psychoanalysis, ecotherapy, and Jungian depth psychology schools were the most prevalent psychological approaches identified in the literature. Psychoanalytic and Jungian authors seemed to converge on interventions that tie the personal to the collective, albeit in slightly different ways. Psychoanalytic authors focused on three elements: ensuring the practitioners were themselves connected to their own experience of climate change, were ready and able to discuss the greater social and systemic ramifications of climate change, and actively connected the client’s personal history to their response to climate change. Jungian authors recommended dreamwork as a tool to connect personal myth and collective myth. Ecotherapy interventions mostly focused on interactions with nature outside of the therapy hour, reflections on the place of nature in clients’ lives and in society within the therapy hour, and cognitive interventions to reframe client’s catastrophic view of climate change, help them pin down the way they can best participate, and engage in meaningful action. Despite these differences, all three psychological schools were united in their shared emphasis on the value of group work as a supportive environment for emotional processing and for connecting one’s inner experience of eco-anxiety to others’ and to greater social themes. Some authors notably referred to established group work models like “The Work that Reconnects” [37] and “Carbon Conversations” [42,55].

### 3.1. Thematic Analysis

The interventions identified in these records were clustered according to five primary themes: fostering clients’ inner resilience, helping clients find social connection and emotional support by joining groups, encouraging clients to take action, practitioner’s inner work and education, and connecting with clients with nature (see Table 1). Table 2 provides further detail on which themes and therapeutic approaches were present in each record.

#### 3.1.1. Theme One: Fostering Clients’ Inner Resilience (*n* = 31 Papers)

This theme clusters interventions aimed at fostering the ability of clients suffering from eco-anxiety to reframe, feel, and make deep meaning out of their distress. These interventions address different levels of the client’s experience (e.g., cognitive, existential, emotional, creative) and revolve around creating strong scaffolding around clients’ inner experience to ensure that emotions can be safely accessed, felt, and expressed.

Sub-Theme: Cognitive Interventions

These interventions focused on identifying and reframing clients’ thoughts, beliefs, and attitudes related to eco-anxiety. Intervention examples include reprioritizing, for example through asking a client, “What’s most urgent? Are there any true emergencies? (What) are (your) skills?” [16] (p. 12) in order to help them establish “their own environmental identity” [16] (p. 12) or inviting them to engage in a thought experiment that asks them to imagine that they “could travel to any other era in history. What would be the great moral or ethical challenge of that era? Every era has its challenges. Would (they) change then for now?” [38] (p. 10).

*Sub-Sub Theme: Shifting from Catastrophizing towards a Less Black and White Picture*

These cognitive interventions specifically focused on helping clients to move out of black-and-white thinking and catastrophizing about climate-related issues and to embrace nuance. Pihkala [7] describes this as the development of “binocular vision” (p. 561), a skill that can enable clients to start accepting that “numerous good and bad things are happening at the same time.”. (p. 561).

Sub-Theme: Meaning-Focused and Existential Interventions

These interventions aimed at helping clients explore how their eco-anxiety impacts key existential concerns in their lives, including their identity and values, the activities they find meaningful and hope-inducing, and their sense of place in the world. Randall [32] in particular argues that even though action steps can be a part of addressing eco-anxiety, they must be preceded by a realistic and empathetic encounter with the existential losses incurred by climate change since “they are likely to be experienced as attacks on the aspects of life that people hold dear: family and attachment, aspiration and progress, individuality, identity, and the self”. (p. 120).

*Sub-Sub-Theme: Discussing and Relativizing the Social and Systemic Dimensions of Climate Change*

Some meaning-focused and existential interventions focused on discussing the client’s place in the world and engaging with the social and systemic dimensions of climate change. These interventions consist in practitioners both providing clients with psychoeducation and normalizing on the collective dynamics of guilt, disavowal and projection that occur in many societies’ handling of the topic of climate change and being able to discuss issues like climate-related structural injustice without assuming that the topic merely reflects a client’s personal neurosis. Practitioners can thus notably explain how unconscious collective guilt about the environment can be split-off and projected by society onto the ecologically aware. Clients could thus be reassured that they may carrying a load that is not just theirs. Lewis [44] explains that it can also be important to support clients who struggle or observe others who struggle with disavowal, defined here as the act of “participating in relatively comfortable fossil-fuel-laden lifestyles while simultaneously knowing about the…suffering wrought by climate change” (p. 24). Providing psychoeducation on the notion of disavowal can be useful and “the therapist should be confirming that the collective enacting of disavowal can be “crazy making”, in that it is confusing and frustrating”. (p. 24).

*Sub-Sub-Theme: Fostering Optimism and Hope*

Other papers in this meaning-focused and existential intervention theme focused on fostering optimism and hope. These interventions are aimed at growing a client’s sense of a realistic and grounded hope. The records within this review that address hope mostly refer to Joanna Macy’s notion of active hope [39,45], “a radical hope that holds steady to the belief that what we can do today is necessary even though we may not know how, when or to whom it will matter” [45] (p. 8), or constructive hope [40].

Sub-Theme: Emotion-Focused Interventions 

These interventions focused on making space for the processing and expression of clients’ emotional experience related to their ecological concerns and on helping them develop emotion regulation skills. The *Coping with Climate Distress* report co-authored by Australian mental health and conservation agencies notably recommends body awareness exercises to individuals to support emotional regulation, as “the more you are aware of your physical body, the more attuned you become to the subtlety of emotion.” [47] (p. 10).

*Sub-Sub-Theme: Grief-Focused Interventions*

Almost half of emotion-focused interventions focused specifically on illuminating and supporting clients’ grief processes inherent to engaging with their eco-anxiety. Baker [35] refers to the need for practitioners to help clients “develop the capacity to move into what (Melanie) Klein termed “the depressive position”, where we are able to experience mourning and loss without recourse to splitting, denial, dissociation or other manic defences” (p. 59). Clients could also be encouraged to relate to their eco-anxiety as a form of anticipatory loss [32].

*Sub-Sub-Theme: Differentiating between Clients’ Distress Related to Their History and Distress Related to Eco-Anxiety*

Two papers that included emotion-focused interventions highlighted the importance of differentiating between client’s distress related to their history and their distress related to eco-anxiety. These interventions aimed at helping the client identify and understand their personal story and trauma and its impact on their response to the issues of climate change, with the goal of supporting their ability to differentiate between them. Lewis [44] provides practitioners with a therapeutic script example to help clients become more aware of the connection between their own experiences of disavowal and society’s manifestations of disavowal. She explains that practitioners could start a conversation by reminding clients that perhaps, in their past, “wrong things were happening, but somehow people both knew and did not know; they knew it but didn’t fully appreciate it enough; they knew it but didn’t take it seriously enough to get help; they knew it but didn’t allow that knowledge in deeply enough to change their behavior. So now you are painfully aware of our lack of full awareness about climate change” (p. 24).

Sub-Theme: Self-Care Interventions 

These interventions focused on helping the client to understand how exhausting engaging with climate change can be and to identify different elements of a self-care protocol to attend to their mental, emotional, and physical needs. In fact, Shaffer [52] quotes a professor explaining that “the first step in combatting ecophobia…is to ensure time to replenish energy” (p. 112). Relevant activities recommended in these five papers include maintaining healthy routines [13,47], practicing mindful self-compassion [51], and practicing gratitude while seeking beauty [48].

Sub-Theme: Interventions Connecting Clients with Their Lyrical Self 

These interventions aimed at exploring client’s creativity and imagination to connect them with different avenues of exploration and self-expression.

*Sub-Sub-Theme: Interventions Focused on Creative Expression and the Arts*

Five papers that included lyrical interventions focused on creative expression and the arts. These interventions relied on verbal and non-verbal creative activities such as visual art, drama therapy and creative writing to help the clients feel into and express their experience of eco-anxiety. Though five papers referenced the general need for interventions involving creative expression, only Pikhala [13] provided tangible examples. He refers to two Finnish art therapy models which have been used to address eco-anxiety, namely participatory drama and facilitated writing workshops.

*Sub-Sub-Theme: Interventions Focused on Dreams*

One intervention in this lyrical sub-theme focused on dreams. This intervention focused on group exploration of clients’ dreams to extract meaning and guidance for the therapeutic process. Gillespie’s [54] mixed-methods study found that group processing of eco-anxiety involving extensive discussion around participants’ dreams helped clients to process difficult emotions and feel more grounded. One participant shared the following takeaway from the experience: “It’s just been profoundly effective in helping me to sort things through... I’ve found it really clarifying and powerful...coming here and just listening to other people’s dreams and having the dreams...feeling safe in this environment has allowed me to make good use of the dreams” [54] (p. 349).

#### 3.1.2. Theme Two: Helping Clients Find Social Connection and Emotional Support by Joining Groups (*n* = 21 Papers)

These interventions were aimed at guiding the client towards supportive groups that are eco-anxiety-informed and specifically intent on supporting the emotional process of individuals suffering from eco-anxiety. Marine biologist Jaramillo [46] makes a poignant plea for the need for such spaces, explaining that, for him, “environmental grief has been the most difficult emotion to deal with” (p. 5), adding: “I don’t know where to go with my grief. Grief is a communal issue. Why would anyone grieve in solitude?” (p. 9). The specific ability of groups to act as strong emotional containers for eco-anxiety-related distress is noted by many papers that include this theme.

Sub-Theme: Joining Established Groups and Organizations 

Papers referring to this theme illustrated the importance of group connection or gave specific recommendations for clients to join established groups and organizations dedicated to supporting individuals suffering from eco-anxiety. Rosemary Randall’s Carbon Conversations groups, referenced in six papers [26,32,33,42,50,55], and Joanna Macy’s The Work that Reconnects groups, referenced in two papers [13,37], are the most prevalent and practitioner-appreciated group models identified in this review. One Carbon Conversations participant highlights the value of its emotional safe space, reflecting that “what Carbon Conversations does that a lot of other organizations or other campaigns don’t do is it actually recognizes and addresses the psychological fear of climate change...it recognizes that it’s a scary fear and maybe that’s why people are burying their heads, and this is a good way of just letting people talk about it” [50] (p. 632). Other group processes mentioned in the literature include Good Grief Network’s eco-anxiety support groups [48], The Dark Mountain Project groups [26], and EcoFaith Recovery’s potlucks [37].

Sub-Theme: Group Rituals 

The three papers touching into this group intervention sub-theme encouraged participation in rituals. Bednarek [53] and Conyer [45] advocated for community grief rituals and Pihkala [7] recommended rituals of the type often offered in faith communities and combining art therapy and rituals. Bednarek [53] shares her rationale behind community grief work, explaining that “if we dare to move through our despair, our heart can break open and become big enough to embrace life in its darkness as well as its richness … but this kind of work requires adequate containment. It is hard to grieve without communal space. We may need to remember old communal practices in this time of collective need” (p. 39).

#### 3.1.3. Theme Three: Encouraging Clients to Take Action (*n* = 15 Papers)

These interventions were aimed at supporting the clients in their efforts to connect to, grow, and enact action plans to reduce their carbon footprint and join collective efforts to defend ecological values.

Sub-Theme: Individual Action 

These interventions were focused on guiding clients towards behavioral changes they can make to align their lifestyles with their environmental values, including eating less meat and dairy and making one’s home energy efficient [43], reducing one’s use of fossil fuels [42], and “extending the limits of one’s comfort zone in different spheres- politically, personally, at work.” [49] (p. 3).

Sub-Theme: Collective Action 

These interventions were focused on guiding clients towards connection with like-minded groups to engage in collective action plans. Although only Burke’s [28] article delved specifically into the collective actions a client might engage with, many records stressed that practitioners could recommend that clients generally join groups focused on active climate change mitigation and adaptation plans. These records also reflected on the ability of group processes to offer an emotional container and safe space that galvanizes participants into action [33,39,41,44]. This rationale could be shared with clients as a piece of psychoeducation.

#### 3.1.4. Theme Four: Practitioner’s Inner Work and Education (*n* = 13 Papers)

This theme refers to interventions the therapeutic practitioner could direct at themselves, namely self-exploration and self-education on the topic of climate change and eco-anxiety. In these 13 records, practitioners are encouraged to explore and understand their own relationship with these themes so that they do not get played out unconsciously in the therapy hour. They are also encouraged to ensure that they are sufficiently trained in how to work with processes related to moving through eco-anxiety, notably trauma and grief. Seaman [26] notably calls for particular attention to the need for practitioners to treat eco-anxiety not as evidence of personal neurosis but rather as evidence of the client being connected to a greater whole.

Sub-Theme: Grief Awareness 

Almost half of the papers touching into the theme of practitioners’ inner work and education refer to grief processes and the specific need for practitioners to educate themselves on various models of grief, including grief as anticipatory loss [32], disenfranchised grief [7] and Worden’s model of the tasks of grief [33] to successfully address eco-anxiety. Randall [32] notably reminds practitioners that “we should not be prescriptive about the kinds of reinvestment of emotional energy we might hope for as people adjust to the losses of climate change… not everyone mourns in the same way.” (p. 123).

#### 3.1.5. Theme Five: Connecting Clients with Nature (*n* = 9 Papers)

These interventions focused on helping clients connect to nature as a space of reflection, resourcing, and inspiration. This included interventions that could take place both in the therapy space, for example, Hasbach’s [36] proposal of expanding the intake interview to include nature-oriented questions, and on the client’s own time. One example is a practitioner’s prescription that clients take regular nature walks and go intentionally into nature without any technology to meditate [16]. Both Dockett [16] and Hasbach [36] also offer clients the possibility of holding sessions outdoors. Most of the interventions offered in this theme revolve around “reawakening our sense of interconnection with the natural world through mindful contact with nonhuman nature, reflective rituals, and other eco-therapeutic techniques such as intentionally engaging in ‘‘earth-caring actions.” [37] (p. 246). 

## 4. Discussion

The review identified interventions for the treatment of eco-anxiety and found the fields of psychoanalysis, ecotherapy, and Jungian depth psychology to be the most prevalent psychological approaches. Only two studies evaluating some of these interventions were available.

These results seem to suggest that treatment for eco-anxiety could embrace a holistic model, reliant on two facets: firstly, evenly addressing different elements and layers of the client’s inner experience and secondly, creating connections between client, practitioner, groups, and nature. In fact, the egalitarian spread of proposed interventions across different microsystems (client and their inner selves, practitioner and their inner selves, client and practitioner, practitioner and professional groups, client and groups, client and nature) mirrors the message contained in many of the papers reviewed, namely that healing both our natural environment and our eco-anxiety must involve a shift away from human and individual-centeredness towards the balanced spreading out of power and attention between the individual, community, and the natural world. Moreover, many of the interventions put forth in this review do not merely focus on helping clients manage their distress but also encourage them to get in touch with something greater than themselves, to access a numinous experience of the world. This is evidenced by the repeated appearance of mindfulness, grief rituals, creative expression, and encouragements to connect with art and literature and to commune with nature [7,12,13,26,37,45,51,52,53]. These are all types of interventions that can bring individuals into deep experiences of grace and stretch the notions of individuality into one of greater systems of connection and healing. It is notable that, in this review, interventions focused solely on connecting clients with nature were present in a quarter of papers (26%), staking nature’s claim as a solid participant in the healing of people.

The high number of proposed interventions centered on the professional’s own reflection on climate change and on adequate training (34%) similarly conveys an important message. It suggests that much like ecologically-oriented action asks every member of the collective to take on their share of responsibility, treatment for eco-anxiety should be democratic and place the practitioner face to face with their own “social and environmental embeddedness” (as cited in Stewart [57], (p. 78)). Seaman’s study [26] further reports that the “therapists’ own emotional reactions to climate change may impact how they receive and respond to clients who discuss climate change during therapy” (p. 46). Many authors emphasize the need for practitioners to make their practice a space in which eco-anxiety can truly be welcomed and not minimized, explained away as a distraction from personal themes, or blocked off because of the professional’s own anxieties [30,32,33,44,45,53]. With regards to training, many papers push for practitioners to acquire more familiarity and skills with regards to both the impacts of climate change and the eco-anxiety process [7,16,26,30,33,42,44,45,53]. Doherty and Clayton [33] postulate that mental health professionals “have an ethical obligation to take immediate steps to minimize harm, reduce disparities in climate impacts, and continually improve their climate-related interventions” [33] (p. 266). Practitioners themselves support this view, as 50% of the therapists interviewed felt that they were not adequately prepared to deal with eco-anxiety, even though 37.3% felt that climate change was relevant both to their work and their field [26].

The review found a high number of proposed interventions involving groups. Unsurprisingly, since mental health interventions often involve connecting the client with inner resources, most records (89%) stressed interventions focused on fostering clients’ inner resilience. However, the degree of presence (62%) of interventions aimed at connecting clients with groups—specifically those groups intent on providing social connection and emotional support rather than groups oriented towards action—is perhaps more surprising. Many group endeavors and organizations focused on actively mitigating climate change have bloomed as public awareness of its consequences has increased (Extinction Rebellion, Plastic Pollution Coalition, among others) but these groups are mostly focused on helping individuals take action. This usually involves making lifestyle changes, engaging politically, or spreading an ecological message. These groups seem to place little emphasis on the emotional experience of feeling distress over our changing climate.

However, this review’s findings reflect a strong theme present across many papers namely that, in order to engage with eco-anxiety, it is just as vital to provide a space for the expression of emotion as it is to act [13,16,26,32,33,37,39,41,45,49,50,55]. The authors cited above seem to concur on a treatment process for eco-anxiety whereby practitioners first provide clients with a safe, containing space in individual or group treatment models in which distressing, and sometimes paradoxical emotions and ideas can be felt and thought through, after which clients can naturally flow into actions that align with their values. Climate anxiety researcher Pikhala [13] and psychoanalyst Randall [32] particularly draw attention to the misguidedness of pushing clients too quickly into action. Pihkala [13] bemoans the oft-adopted model that “the antidote to anxiety is action” (p. 11) and Randall explains that a focus on guiding clients towards ecological lifestyles changes without first addressing the immense losses and attacks on identity those changes will catalyze is doomed to fail [32].

According to this review, established groups and organizations have a crucial role to play in providing this supportive collective environment. Randall’s Carbon Conversations group model and Joanna Macy’s The Work that Reconnects are appraised in consistently laudatory terms by authors [13,26,33,37,42,50,55] and by participants, in great part because of their capacity to provide an emotional safe space for participants. These two models could therefore function as blueprints for group treatment of eco-anxiety.

One of the reasons that group interventions appear so much in the literature reviewed, according to many authors, is because they can act as powerful emotional containers for the profound, existential distress that tends to accompany eco-anxiety. This review identifies grief as the most central emotional component of this distress, as 14 of the 34 records reviewed pointedly refer to it. In doing so, they seem to validate the definitions of eco-anxiety proposed by mental health practitioner Lertzman (as cited by Dockett [16]) and researcher Albrecht [17], who conceive of eco-anxiety as form of deep mournful melancholia. These 14 records also highlight the need for mental health professionals treating eco-anxiety to be familiar with the general process of grief, with authors especially concurring on the value of Worden’s model of the tasks of grief. The Good Grief Network [48] and Randall [49] also offer their own multi-step blueprints for moving through the experience. The presence of these models seems to cement the conceptualization of eco-anxiety as a process through which an individual can be accompanied.

The 14 records equally point to the importance of understanding the specific kinds of grief underlying eco-anxiety. Two authors [10,13] notably offer their proposal that eco-anxiety is a form of disenfranchised grief, thus requiring both that practitioners be familiar with the concept and that they be prepared to offer the required heightened level of support that must accompany a grief that is deemed not socially acceptable. Fittingly, three authors recommend group mourning rituals [7,45,53] in an effort to spread the weight of the grief process and to provide participants with the experience of being seen and validated by the collective in their pain. The literature identified in this review repeatedly highlights the tension between the individual and the collective, namely the line betweenwhat is personal pain and responsibility, and what can and should be shouldered by a greater group of peers.

Four authors identified in the review [13,16,42,53] refer to the traumatic component of eco-anxiety, with Bednarek [53] and Pihkala [13] specifically confirming Van Susteren (as cited in Kerecman Myers [22]) and Kaplan’s [23] conceptualization of eco-anxiety as a form of anticipatory trauma. However, these authors focus on the need for practitioners to familiarize themselves with this kind of trauma rather than proposing specific, trauma-informed interventions. This illustrates a larger finding of the review, namely a great disparity in the level of specificity of interventions proposed across the records reviewed. Some interventions are highly targeted, for example Randall’s recommendation that mental health professionals provide psychoeducation on the dynamics of unconscious collective guilt [30] or Hasbach’s ecotherapy intervention whereby clients identify a space in nature that they like and visit it regularly to build a nurturing relationship with it [36]. Other interventions are far more general, as evidenced by Davenport’s recommendation that clients should be encouraged to cultivate community connection [10] or the Good Grief Network’s vague exhortation to eco-anxious individuals that they “do inner work” [48] (p. 3). This disparity could be due to two factors. First, more than half of the records reviewed take a holistic approach to eco-anxiety, proposing many interventions that address the subject from a variety of different angles rather than exploring a few interventions in depth. Second, most of the records identified in the review are reflections on the general subject of eco-anxiety which happen to mention interventions rather than papers or studies pointedly addressing or measuring treatment interventions for eco-anxiety.

The dearth of studies evaluating any of the proposed interventions highlights a major gap in the literature. Only two studies were identified that evaluated interventions. Gillespie’s [54] study evaluated a dreamwork-oriented group process and Büchs et al.’s [50] study assessed the effects of participating in Carbon Conversations groups. The encouraging results of these studies reaffirm the value of some of the interventions proposed by authors, most of all the power of groupwork, and make a strong case for increasing the evaluation of other, yet-unassessed treatment possibilities to create a strong, evidence-based foundation. These two studies also shared a compelling finding. Aside from helping participants to process complex and sometimes contradictory emotions and to gain insight and grounding from others’ perspectives, both Gillespie’s group and Büchs, Hinton and Smith’s Carbon Conversation sample found that the group process had positively affected participants’ ability and willingness to take tangible, ecologically-minded action. Indeed, Gillespie [54] explains that “as a result (of the group process) we felt better equipped and more motivated to work with others to engage with global warming issues” (p. 353). Büchs et al. [50] “found evidence that …Carbon Conversations…has helped many participants to engage with these issues more deeply and take- sometimes quite radical- carbon reducing actions in multiple areas” (p. 636) and that “several participants also report that taking part made them feel more confident in talking to and encouraging people in their personal networks to reduce their emissions, indicating that the impact of Carbon Conversations may extend beyond the direct participants” (p. 636). This shared finding could indicate that treating eco-anxiety through providing its sufferers with shared emotional space may have far greater impacts than merely helping individuals better tolerate their distress. It could also be an important part of helping people change their lives in a way that goes beyond the scope of mere treatment and shifts their relationship with the world. A wealth of literature is available on the complex topic of getting people to change their habits in line with sustainability goals, and studies like these could act as the bridge between strict behavioral conceptions of change and deeper, more holistic, and emotion-embracing approaches.

### Strengths and Limitations

The most apparent strength of this review may be that it is the first scoping review of this topic, in an era where its importance and relevance are clear. Other strengths included the thorough and extensive database searching and broad inclusion criteria. The latter point proved to be a limitation as well as a strength. Our effort to be broadly inclusive was necessary to address the scant nature of articles published on the topic, however, this led to the inclusion of records that were reflections on eco-anxiety with brief references to interventions, rather than literature targeted on specific interventions. Another limitation was the inability to access certain records whose abstracts suggested they would be highly relevant. The last limitation of this review was the decision not to include books or book chapters, or literature in languages other than English. The identification of many books and book chapters addressing interventions for eco-anxiety during the selection process suggests that the inclusion of these materials in future reviews could provide rich insight. This scoping review inherently focused on providing readers with breadth and comprehensiveness into the topic, and as such, future studies could pay more attention to depth of analysis and validity assessment.

## 5. Conclusions

The review identified a variety of interventions for both individual and group treatment. These interventions targeted many layers of individuals’ wellbeing, from inner experiences such as thought processes to connecting with others through sharing and rituals and to communing with the natural world. Recommendations for treatment plans are to focus on holistic, multi-pronged, and grief-informed approaches that include eco-anxiety-focused group work.

## Figures and Tables

**Table 1:** Eco-anxiety themes.

Main Themes	*N* *	Sub Themes	Sub-Sub Themes
Fostering clients’ inner resilience.	31	Cognitive interventions.	Shifting from catastrophizing towards a less black-and-white picture.
		Meaning-focused and existential interventions.	Discussing and relativizing the social and systemic dimensions of climate change.
			Fostering optimism and hope.
		Emotion-focused interventions.	Grief-focused interventions.
			Differentiating between clients’ distress related to their history and distress related to eco-anxiety.
		Self-care interventions.	
		Interventions connecting clients with their lyrical self.	Interventions focused on creative expression and the arts.
			Interventions focused on dreams.
Helping clients find social connection and emotional support by joining groups.	21	Joining established groups and organizations.	
		Group rituals.	
Encouraging clients to take action.	15	Individual action. Collective action	
Practitioner’s inner work and education.	13	Grief awareness.	
Connecting clients with nature	9		

* *N* = number of articles/papers.

**Table 2:** Intervention themes, sub-themes, and sub-sub themes identified in scoping review.

Author, Date	Approach	Themes Identified
Conyer [45]	Counselling, narrative therapy	Fostering clients’ inner resilience: meaning-focused/existential interventions—fostering optimism and hope; interventions connecting clients with their lyrical self—interventions focused on creative expression and the arts. Helping clients find social connection and emotional support by joining groups: rituals. Encouraging clients to take action. Practitioner’s inner work and education. Connecting clients with nature.
Clayton [41]	Conservation psychology	Fostering clients’ inner resilience: cognitive interventions. Helping clients find social connection and emotional support by joining groups. Encouraging clients to take action: collective action.
White [55]	Ecopsychology	Helping clients find social connection and emotional support by joining groups: joining established groups and organizations. Encouraging clients to take action.
Dockett [16]	Includes ecotherapy	Fostering clients’ inner resilience: cognitive interventions—shifting from catastrophizing towards a less black and white picture; emotion-focused interventions. Encouraging clients to take action: individual action and collective action. Practitioner’s inner work and education. Connecting clients with nature.
Hasbach [36]	Ecotherapy	Connecting clients with nature.
Koger [37]	Ecotherapy	Helping clients find social connection and emotional support by joining groups: joining established groups and organizations. Connecting clients with nature.
Kelsey [56]	Environmental education	Fostering clients’ inner resilience: cognitive interventions—shifting from catastrophizing towards a less black and white picture.
Bednarek [53]	Gestalt	Helping clients find social connection and emotional support by joining groups: rituals. Practitioner’s inner work and education. Connecting clients with nature.
Gillespie [54] *	Jungian depth psychology	Fostering clients’ inner resilience: interventions connecting clients with their lyrical self—interventions focused on dreams. Helping clients find social connection and emotional support by joining groups.
Kiehl [34]	Jungian depth psychology	Fostering clients’ inner resilience: meaning-focused/existential interventions.
Davenport [10]	Marriage and family therapy	Fostering clients’ inner resilience: meaning-focused/existential interventions. Helping clients find social connection and emotional support by joining groups. Practitioner’s inner work and education: grief awareness.
Baker [35]	Psychoanalysis	Fostering clients’ inner resilience: emotion-focused interventions.
Haseley [42]	Psychoanalysis	Fostering clients’ inner resilience: emotion-focused interventions—differentiating between distress related to the client’s history and distress due to eco-anxiety. Helping clients find social connection and emotional support by joining groups: joining established groups and organizations.
Lewis [39]	Psychoanalysis	Fostering clients’ inner resilience: meaning-focused/existential interventions—fostering optimism and hope; emotion-focused interventions. Helping clients find social connection and emotional support by joining groups. Encouraging clients to take action: collective action.
Lewis [44]	Psychoanalysis	Fostering clients’ inner resilience: meaning-focused/existential interventions—discussing and relativizing the social and systemic dimensions of climate change; emotion-focused interventions—differentiating between distress related to the client’s history and distress due to eco-anxiety. Encouraging clients to take action: collective action. Practitioner’s inner work and education.
Randall [30]	Psychoanalysis	Fostering clients’ inner resilience: meaning-focused/existential interventions—discussing and relativizing the social and systemic dimensions of climate change. Practitioner’s inner work and education.
Randall [32]	Psychoanalysis	Fostering clients’ inner resilience: emotion-focused interventions—grief. Helping clients find social connection and emotional support by joining groups: joining established groups and organizations. Practitioner’s inner work and education: grief awareness.
Randall [49]	Psychoanalysis	Fostering clients’ inner resilience: meaning-focused/existential interventions, emotion-focused interventions—grief. Encouraging clients to take action: individual and collective action. Practitioner’s inner work and education: grief awareness.
Australian Conservation Foundation [47] **	Not identified	Fostering clients’ inner resilience: cognitive interventions; meaning-focused/existential interventions—fostering optimism and hope; emotion-focused interventions, self-care interventions. Helping clients find social connection and emotional support by joining groups. Encouraging the client to take action: individual action. Connecting clients with nature.
Büchs et al. [50] *	Not identified	Helping clients find social connection and emotional support by joining groups: joining established groups and organizations.
Burke [38]	Not identified	Fostering clients’ inner resilience: cognitive interventions—shifting from catastrophizing towards a less black and white picture; emotion-focused interventions. Encouraging clients to take action: individual action and collective action.
Clayton et al. [15] **	Not identified	Fostering clients’ inner resilience: cognitive interventions; meaning focused/existential interventions. Helping clients find social connection and emotional support by joining groups.
Doherty and Clayton [33]	Not identified	Fostering clients’ inner resilience: emotion-focused interventions—grief. Encouraging clients to take action: individual and collective action. Practitioner’s inner work and education: grief awareness.
Good Grief Network [48] *	Not identified	Fostering clients’ inner resilience: cognitive interventions; meaning-focused/existential interventions; self-care interventions. Encouraging clients to take action.
Hayes [12] *	Not identified	Fostering clients’ inner resilience: cognitive interventions, interventions connecting clients with their lyrical self—interventions focused on creative expression and the arts. Helping clients find social connection and emotional support by joining groups. Connecting clients with nature.
Jaramillo [46]	Not identified	Fostering clients’ inner resilience: emotion-focused interventions—grief.
Kelly [51] *	Not identified	Fostering clients’ inner resilience: cognitive interventions; emotion-focused interventions; self-care interventions. Helping clients find social connection and emotional support by joining groups. Encouraging clients to take action. Connecting clients with nature.
Ojala [40]	Not identified	Fostering clients’ inner resilience: meaning-focused/existential interventions—fostering optimism and hope and discussing/relativizing the social and systemic dimensions of climate change. Encouraging clients to take action: collective action.
Pihkala [7]	Not identified	Fostering clients’ inner resilience: cognitive interventions— shifting from catastrophizing towards a less black and white picture; meaning-focused/existential interventions; interventions connecting clients with their lyrical self—interventions focused on creative expression and the arts. Helping clients find social connection and emotional support by joining groups: rituals. Practitioner’s inner work and education: grief awareness.
Pikhala [13]	Not identified	Fostering clients’ inner resilience: cognitive interventions; emotion-focused interventions—grief; self-care interventions; interventions connecting clients with their lyrical self—interventions focused on creative expression and the arts. Helping clients find social connection and emotional support by joining groups: joining established groups and organizations. Encouraging the client to take action-individual action. Practitioner’s inner work and education.
Sarchet [43] **	Not identified	Fostering clients’ inner resilience: emotion-focused interventions. Helping clients find social connection and emotional support by joining groups. Encouraging clients to take action: individual action. Connecting clients with nature.
Seaman [26] *	Not identified	Fostering clients’ inner resilience: cognitive interventions; meaning-focused/existential interventions—discussing and relativizing the social and systemic dimensions of climate change, emotion-focused interventions—grief focused interventions. Helping clients find social connection and emotional support by joining groups—joining established groups and organizations. Practitioner’s inner work and education.
Shaffer [52]	Not identified	Fostering clients’ inner resilience: self-care interventions; interventions connecting clients with their lyrical self—interventions focused on creative expression and the arts. Helping clients find social connection and emotional support by joining groups.
Stokols et al. [31]	Not identified	Helping clients find social connection and emotional support by joining groups.

* Empirical studies, ** instructions directly targeted at clients.

## Appendix A

### Search Terms

Eco-anxiety; Eco anxiety; Ecoanxiety; Ecoanxiety treatment; Eco-anxiety treatment
Eco anxiety treatment; Eco anxiety intervention; Ecological anxiety treatment
Environmental anxiety; Eco angst; Climate change distress; Climate distress treatment; Climate distress intervention; Climate change fear
